# The Small GTPase RhoA Is Required for Proper Locomotor Circuit Assembly

**DOI:** 10.1371/journal.pone.0067015

**Published:** 2013-06-25

**Authors:** Shalaka Mulherkar, Feng Liu, Qin Chen, Anjana Narayanan, Anthony D. Couvillon, H. David Shine, Kimberley F. Tolias

**Affiliations:** 1 Department of Neuroscience, Baylor College of Medicine, Houston, Texas, United States of America; 2 Center for Cell and Gene Therapy, Baylor College of Medicine, Houston, Texas, United States of America; 3 Verna and Marrs McLean Department of Biochemistry and Molecular Biology, Baylor College of Medicine, Houston, Texas, United States of America; 4 Department of Molecular and Cellular Biology, Baylor College of Medicine, Houston, Texas, United States of America; 5 Department of Medicine, Beth Israel Deaconess Medical Center, Harvard Medical School, Boston, Massachusetts, United States of America; Institut de la Vision, France

## Abstract

The assembly of neuronal circuits during development requires the precise navigation of axons, which is controlled by attractive and repulsive guidance cues. In the developing spinal cord, ephrinB3 functions as a short-range repulsive cue that prevents EphA4 receptor-expressing corticospinal tract and spinal interneuron axons from crossing the midline, ensuring proper formation of locomotor circuits. Here we report that the small GTPase RhoA, a key regulator of cytoskeletal dynamics, is also required for ephrinB3/EphA4-dependent locomotor circuit formation. Deletion of RhoA from neural progenitor cells results in mice that exhibit a rabbit-like hopping gait, which phenocopies mice lacking ephrinB3 or EphA4. Consistent with this locomotor defect, we found that corticospinal tract axons and spinal interneuron projections from RhoA-deficient mice aberrantly cross the spinal cord midline. Furthermore, we determined that loss of RhoA blocks ephrinB3-induced growth cone collapse of cortical axons and disrupts ephrinB3 expression at the spinal cord midline. Collectively, our results demonstrate that RhoA is essential for the ephrinB3/EphA4-dependent assembly of cortical and spinal motor circuits that control normal locomotor behavior.

## Introduction

In the developing nervous system, proper neuronal circuit formation depends on the accurate navigation of axons, which are guided to their target cells by attractive and repulsive cues. Short-range repulsive axon guidance decisions are mediated by Eph receptor tyrosine kinases and their membrane-bound ephrin ligands [1], [2]. For instance, genetic studies in mice have demonstrated that EphA4 and ephrinB3 cooperate to regulate repulsive axon guidance during locomotor circuit assembly. Mice lacking EphA4, ephrinB3, or EphA4 kinase activity display a striking motor defect characterized by synchronous hindlimb movements and an abnormal rabbit-like hopping gait [3]–[6]. This locomotor defect is caused by the inappropriate midline crossing of corticospinal tract (CST) and spinal interneuron axons, which results in aberrant contralateral spinal cord innervation [3]–[5]. Ordinarily, EphA4-expressing axons are prevented from crossing the spinal cord midline by a repulsive ephrinB3 midline barrier [4], [7].

Eph receptors are thought to regulate axon guidance by modulating the activity of Rho GTPases [8], [9]. Rho GTPases are key regulators of the cytoskeleton that play essential roles in controlling the development and remodeling of the central nervous system (CNS) [10]. For instance, Rac1 promotes the growth and branching of axons and dendrites and the formation and maintenance of excitatory synapses, whereas RhoA inhibits these processes [11]–[14]. Rho GTPases also regulate cell-cycle progression, survival, polarization, migration, and cell-cell adhesion [8], [9]. The ability of Rho GTPases to regulate these diverse cellular processes is facilitated by guanosine nucleotide exchange factors (GEFs) and GTPase-activating proteins (GAPs), which activate or inhibit Rho GTPases, respectively, in response to extracellular stimuli [15]. Several Rho family regulatory proteins have been identified as mediators of EphA4-dependent axon guidance [16]–[19]. In particular, the Rac-GAP α2-Chimaerin has been shown to function as a downstream effector of EphA4 that is essential for EphA4-dependent axon navigation during the assembly of locomotor neural circuits [20]–[22].

Much of our understanding of mammalian Rho GTPase function comes from *in vitro* studies using dominant-negative and constitutively-active mutants or pharmacological inhibitors. To further elucidate the *in vivo* functions of RhoA in the developing CNS, we employed a genetic approach to conditionally ablate RhoA in neural progenitor cells. Anatomical and behavioral characterization of these mice revealed phenotypes similar to mice lacking EphA4-dependent forward signaling. Specifically, RhoA-deficient mice exhibit aberrant wiring of neuronal circuits that control coordinated limb movements, resulting in an abnormal rabbit-like hopping gait. These defects may be caused by a failure of EphA4-expressing axons to be repulsed by ephrinB3 and/or the aberrant expression of ephrinB3 at the spinal cord midline. These findings demonstrate an essential and nonredundant role of RhoA in the ephrinB3-EphA4-dependent assembly of neuronal locomotor circuits.

## Materials and Methods

### Generation of Conditional Knock-out Mice

**Figure 1:**
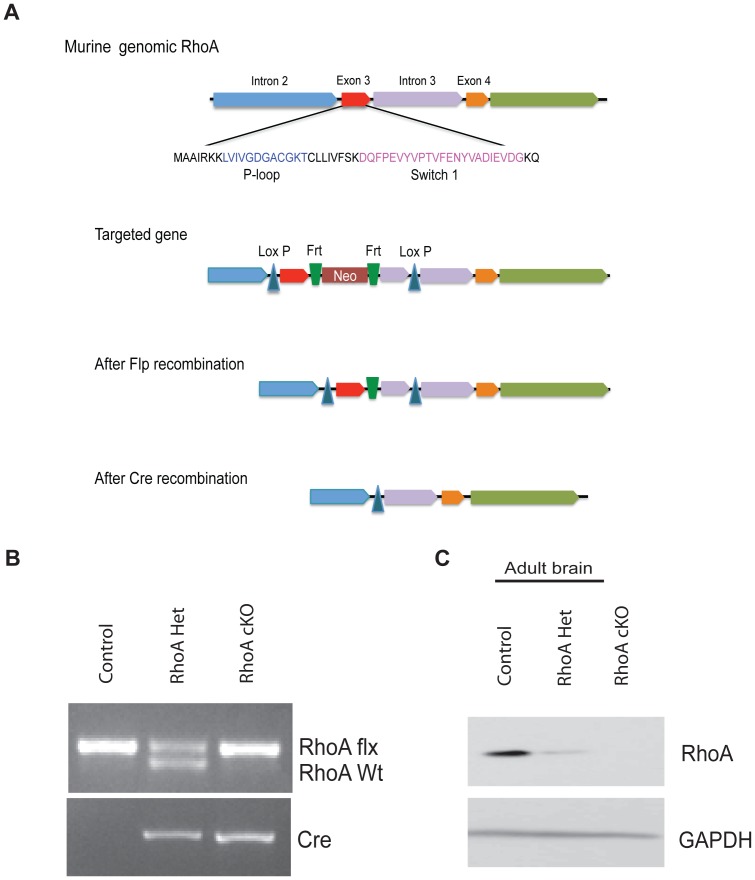
Generation and characterization of mice containing a conditional allele of*RhoA.* (A) Schematic diagram of the strategy used to generate RhoA conditional knockout mice. A conditional *RhoA* allele was created by inserting two LoxP sites in a region of the *RhoA* gene flanking exon 3 and part of intron 3 (*RhoA^fl/fl^*). An internal Frt-flanked neomycin (Neo) casette was also introduced as a selection marker, which was subsequently removed by crossing mutant mice with mice expressing Flippase (Flp recombination). The region of the *RhoA* gene between the two loxP sites was then excised in neuroprogenitor cells by crossing the *RhoA^fl/fl^* mice with Nestin-Cre mice (Cre recombination). (B) PCR genotyping of *RhoA^fl/fl^* (control), *RhoA^fl/+^;Nestin-Cre* (RhoA het), and *RhoA^fl/fl^;Nestin-Cre* (RhoA cKO) mice. Top gel, *RhoA* allele; bottom gel, *Cre*. (C) Protein isolated from the brains of adult control, RhoA het, and RhoA cKO mice were immunoblotted with antibodies against RhoA or GAPDH (loading control) to assess loss of RhoA expression.

A conditional *RhoA* allele, in which exon 3 and part of intron 3 are flanked by two loxP sites (*RhoA^fl/fl^*), was generated as depicted in Fig. 1A. Prior to conducting experiments, the Neo cassette was removed by crossing the mice with Flippase-expressing mice. *RhoA^fl/fl^* mice were then crossed with *RhoA^fl/+^;Nestin-Cre* (RhoA het) mice to obtain *RhoA^fl/fl^;Nestin-Cre* (RhoA cKO) as well as RhoA het and *RhoA^fl/fl^* (control) littermates for experiments. Genotyping was performed by polymerase chain reaction (PCR).

### Ethics Statement

All animal experiments were carried out in strict accordance with the recommendations provided by the Guide for the Care and Use of Laboratory Animals of the National Institutes of Health and were approved by the Baylor College of Medicine Institutional Animal Care and Use Committee (AN-4365). All efforts were made to minimize animal suffering.

### Antibodies

The following purchased antibodies were used in our study: rabbit polyclonal antibodies anti-RhoA, anti-RhoB, and anti-RhoC (Cell Signaling Technology, Beverly, MA); mouse monoclonal anti-EphA4 receptor and rabbit polyclonal anti-ephrinB3 (Invitrogen, Camarillo, CA); anti-GAPDH antibody (Millipore, Temecula, CA); and the mouse monoclonal anti-acetylated tubulin antibody (Sigma, St. Louis, MO). For secondary antibodies, we used goat polyclonal antibodies labeled with horseradish peroxidase for Western blotting or with Cy3 or AlexaFluor 488 for immunostaining (Jackson ImmunoResearch Laboratories, West Grove, PA). Alexa 594-labeled phalloidin was purchased from Molecular Probes (Eugene, OR).

### Protein Lysates and Western Blot Analysis

**Figure 2:**
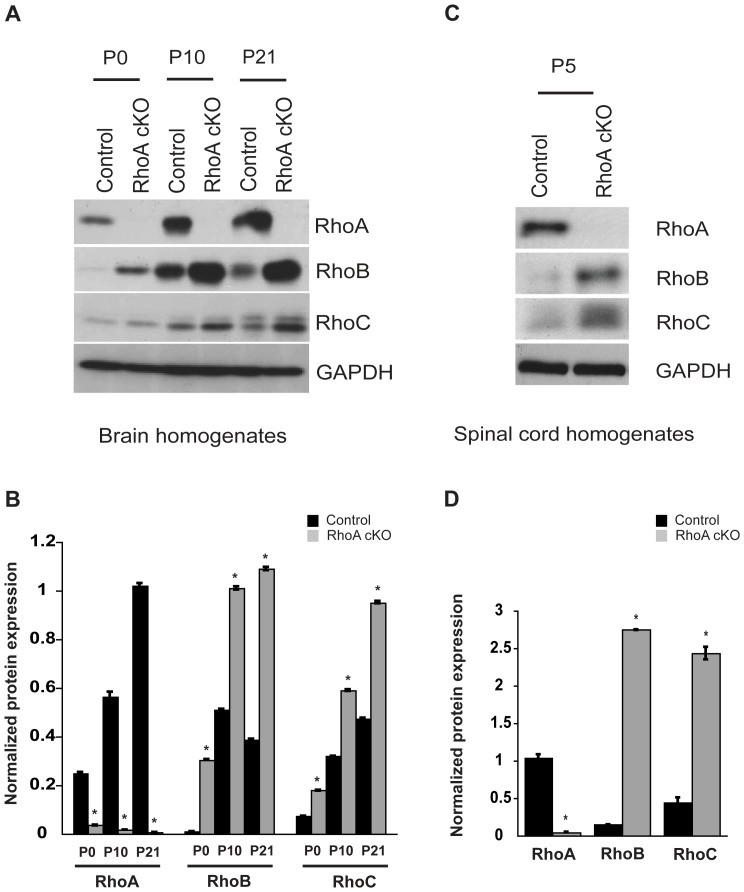
Expression of Rho isoforms in the developing mouse brain and spinal cord. Brain (A) and spinal cord (C) homogenates prepared from control and RhoA cKO mice at different developmental ages were immunoblotted for the different Rho isoforms (RhoA, RhoB and RhoC) or GAPDH (loading control). Each blot is representative of 3 independent experiments. (B) Normalized levels of RhoA, RhoB and RhoC expression in the mouse brain at different developmental stages (P0, P10, P21). Protein bands from three independent experiments were quantified using NIH Image J software and normalized using GAPDH as control. Data are represented as mean ±S.E.M. (*p<0.001, Student’s t-test). (D) Quantification of different Rho isoforms (RhoA, RhoB, RhoC) from P5 mouse spinal cord was performed as stated in Fig. 2B. Data are represented as mean ±S.E.M. from three independent experiments (*p<0.001, Student’s t-test).

Primary neurons were lysed in NP-40 lysis buffer (50 mM Tris pH 7.5, 150 mM NaCl, 1 mM EDTA, 1%; NP-40, 5% glycerol) containing protease inhibitors (complete mini cocktail, Roche, Mannheim, Germany) and phosphatase inhibitors for immunoblot analysis. Brain and spinal cord tissue were homogenized in NP-40 lysis buffer using a Dounce homogenizer. Protein amounts were estimated using Bradford reagent (Biorad, Carlsbad, CA). Cell lysates were separated using sodium dodecyl sulfate (SDS) –polyacrylamide gel electrophoresis and transferred onto PVDF membranes. The membranes were incubated in blocking buffer [5% skimmed milk powder in 50 mM Tris, pH 8.0, 150 mM NaCl, 0.05% Tween (TBST)] followed by an overnight incubation with primary antibody at 4°C. Horseradish-peroxidase-labeled secondary antibody binding was detected by enhanced chemiluminescence (ECL, Pierce, Rockford, IL). Autoradiography films (VWR International, Radnor, PA) were scanned and the bands were quantified using Image J software (Fig. 2B, D).

### Gait Analysis

**Figure 3:**
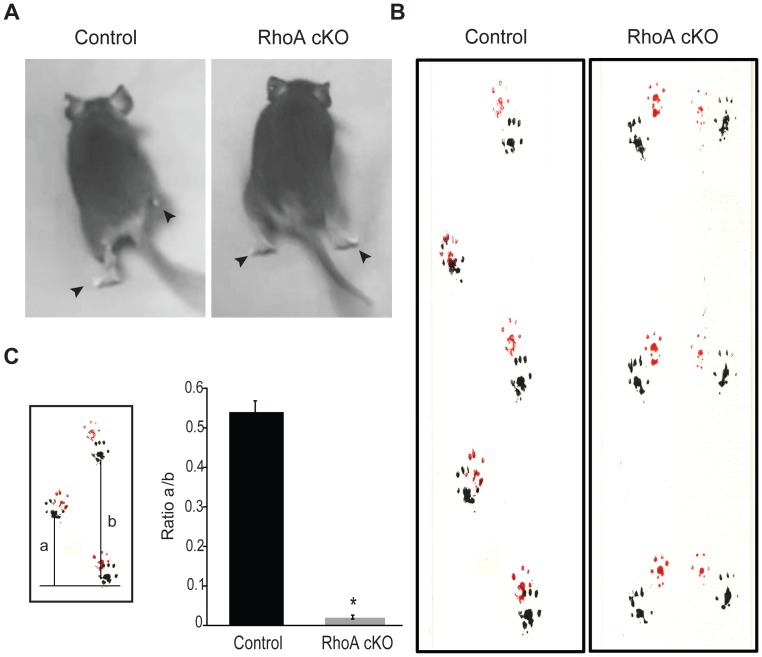
RhoA cKO mice display a rabbit-like hopping gait. (A) Control littermates display alternate limb movements, while RhoA cKO mice show a highly abnormal synchronous gait. Arrowheads indicate the position of hindlimbs with respect to each other. (B) Gait analysis of control and RhoA cKO mice. Non-toxic paint was applied to the forepaws (red) and hindpaws (black) of mice, and then they were allowed to walk on a piece of white paper to record the placement pattern of their footprints. (C) Determination of the distance between the right and left paw (a) and the distance between the same paw (b) to assess the degree of parallel movement of the limbs (comparison of a/b ratios). Data are represented as the mean ± S.E.M. (N = 6 animals per genotype), *p<0.001 (Student’s t-test).

A 40 cm long, 5 cm wide runway was lined with white paper at the bottom. Mice were trained to run down the runway in a straight line a day before the test. On the test day, the paws of the mice were dipped in non-toxic acrylic paint (forepaws red, hindpaws black) and mice were allowed to run down the runway. The footprints were analyzed for interstep distance (the distance between the placement of the right hindpaw or forepaw and the placement of the left hindpaw or forepaw, Fig. 3C-a) and intrastep distance (the distance covered by the same hindpaw or forepaw, Fig. 3C-b) to determine the degree of parallel movement of the limbs.

### Anterograde Tracing and Analysis of the Corticospinal Tract

**Figure 4:**
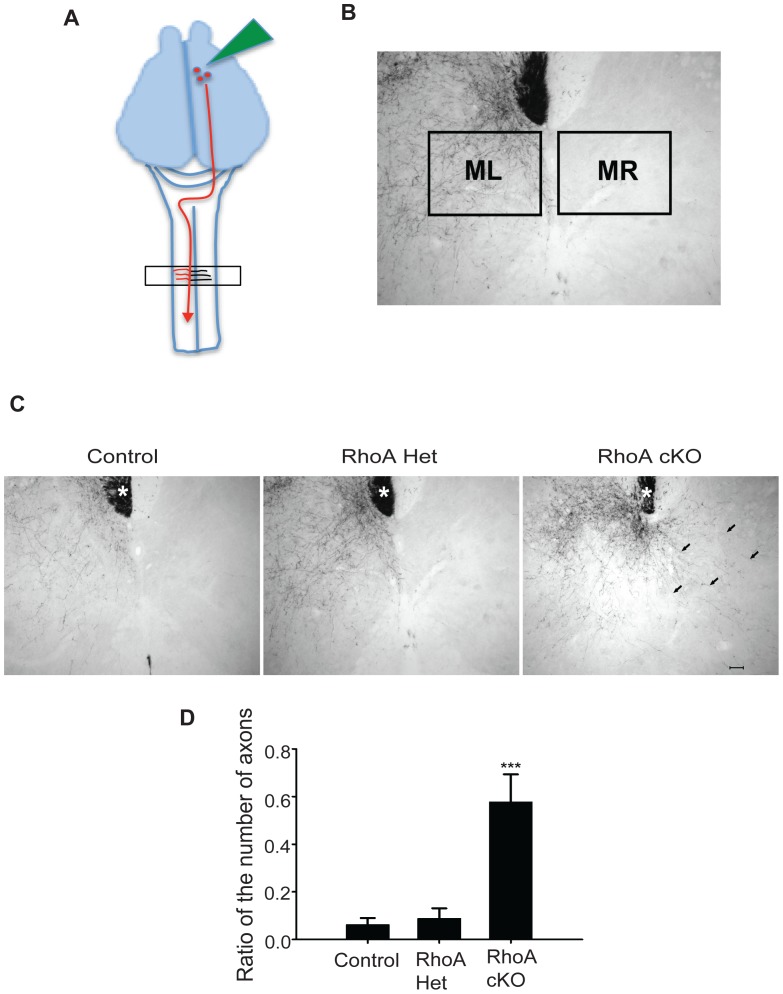
Aberrant midline crossing of corticospinal tract axons in RhoA cKO mice. (A) Schematic diagram showing the strategy used to label CST axons. (B) Photomicrograph of the cervical spinal cord showing the two regions (ML and MR) that were analyzed for the presence of labeled axons. The value of axons in the region contralateral to the labeled CST was expressed as the ratio of the number of pixels in the region MR to the number of pixels in the region ML. (C) Sections from control, heterozygous, and RhoA cKO mice showed that most CST axons exited from the CST within the dorsal funiculi (marked with asterisks) and projected into the gray matter ipsilateral to the labeled CST in control, heterozygous, and RhoA cKO mice. However, in the RhoA cKO mice only, many BDA-labeled CST axons (arrows) were observed projecting into the gray matter contralateral to the labeled CST. Scale bar: 50 µm. (D) The ipsilateral/contralateral ratio of the numbers of labeled CST axons in the gray matter of the cervical spinal cords in RhoA cKO was significantly greater than that of heterozygous and control mice. N = 3–4 mice/group. Data are represented as the mean ± SD. ***p<0.001. (ANOVA followed by the Student-Newman-Keuls test).

CST axons were labeled with biotinylated dextran amine (BDA; lysine fixable, MW 10,000; Molecular Probes, Eugene, OR) using a method previously described [23]. Mice were anesthetized with isoflurane, positioned in a stereotaxic frame, and the right sensorimotor cortices exposed. A solution of BDA (10% in phosphate buffered saline (PBS), pH 7.4) was injected using a Nanoliter injector (World Precision Instruments, Sarasota, FL) into 8 sites (220 nl per site) in two rows of 4 sites in the right sensorimotor cortices that project to regions of the spinal cord that innervate the forelimbs and hindlimbs [24]. The mice were sacrificed nine days after the BDA injection. The cervical regions of the spinal cord (C1–C7) were removed, postfixed for 6 hrs and then infiltrated with 21% sucrose in PBS for cryoprotection. Cross sections (40 µm) were cut on a cryostat and stored at –20°C until they were processed for histochemistry. The BDA labeled axons were visualized with avidin-biotin complexes reagent (Vector Laboratories, Burlingame, CA) and diaminobenzidine solution (0.7 mg/ml) containing 0.06% nickel chloride and 0.015% hydrogen peroxide. The number of axons was determined by an investigator who was unaware of the genotype of each animal. The quantity of axons was measured from the photomicrographs using NIH Image J software (NIH, Bethesda, MD) and the FeatureJ plug-in as described previously [25], [26]. The number of axons was measured as the number of pixels in the region (Fig. 4B, MR) contralateral to the labeled CST. To avoid error resulting from variable BDA labeling efficiencies among mice, the number of pixels in a corresponding region (Fig. 4B, ML) in the gray matter ipsilateral to the labeled CST was also measured. The degree of axons that innervated the spinal cord contralateral to the labeled CST was expressed as the ratio of the number of pixels in the region MR to the number of pixels in the region ML.

### Measurement of Dorsal Column and Gray Matter

**Figure 5:**
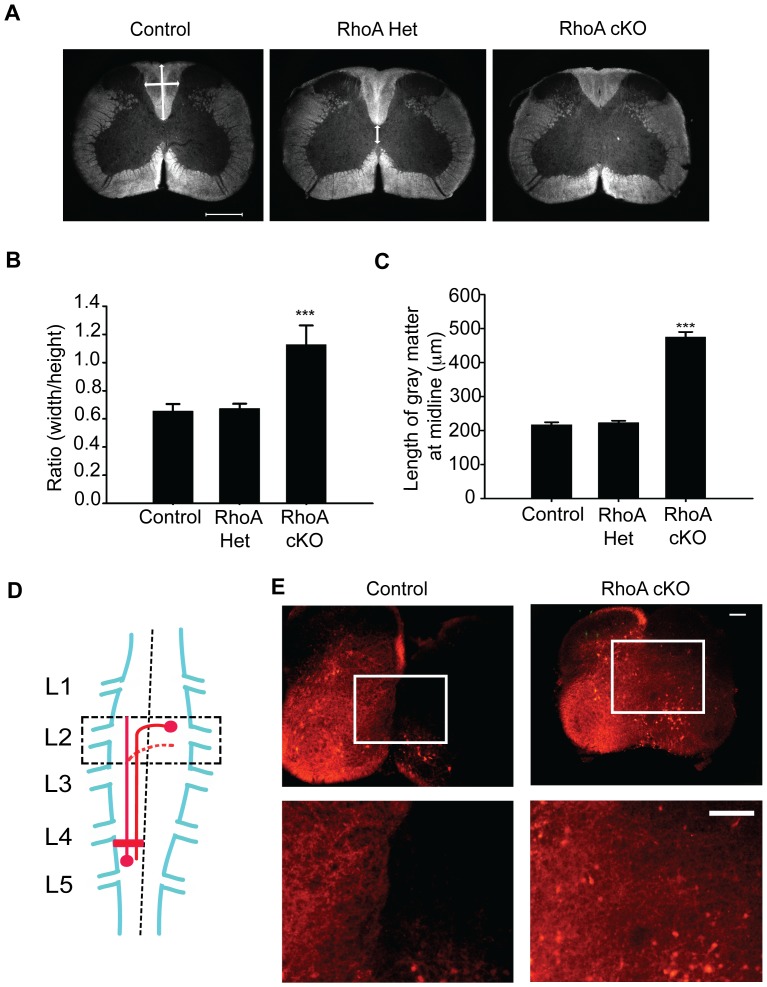
Abnormal morphology and incorrect innervation in the spinal cord of RhoA cKO mice. (A) Dark field photos demonstrated that the dorsal funiculi of RhoA cKO mutant mice at the cervical spinal cord were more shallow and widened compared to the control and heterozygous mice. RhoA cKO mice exhibited a remarkable increase in the amount of gray matter at the midline. The width (horizontal line in A, left panel) and the height (vertical line in A, left panel) of funiculi were measured. Scale bar: 500 µm. (B) The ratio of width to height measurements for the dorsal funiculus of RhoA cKO mice was significantly greater than that of control and heterozygous mice (p<0.001). No significant differences were observed between control and heterozygous mice (p>0.05) (C). At the midline, the space between the dorsal column and ventral column of gray matter of RhoA cKO mice was significantly greater compared to control and heterozygous mice (p<0.001). n = 3–4 mice per group. Data are represented as the mean ± SD. *** = *p*<0.001 (ANOVA followed by the Student-Newman-Keuls test). (D) Schematic drawing illustrating the strategy used for labeling spinal interneurons. Crystals of rhodamine dextran were applied unilaterally to control and RhoA cKO isolated mouse spinal cords at L4 (red rectangle). Contralateral projections were then visualized by imaging the labeled spinal cords at L2 (black dashed box) with an epifluorescence microscope. (E) Many spinal interneuron axons aberrantly cross the midline at L2 in the spinal cords of RhoA cKO mice, but not control littermates. Lower panels show an enlarged image of the area boxed in the upper panels. Scale bar represents 200 µm. Three sections per animal and N = 4 animals were analyzed per genotype.

To compare the morphologic change of dorsal column, the height (vertical line in figure 5A, left panel) and width (horizontal line in figure 5A, left panel) of dorsal column at the cervical level were measured and the ratio of width to height was calculated. The space between bottom of dorsal column and the top of ventral column of gray matter at midline (vertical line in Fig. 5A, middle panel) was also measured.

### Labeling of Spinal Interneurons

The spinal interneurons were traced as previously described [20] with some modifications. Briefly, postnatal day 5 (P5) mice were deeply anaesthetized with isoflurane and decapitated. Spinal cords were dissected out in cold artificial cerebrospinal fluid (aCSF) (aCSF: 125 mM NaCl, 2.5 mM KCl, 1.25 mM NaH_2_PO_4_, 25 mM NaHCO_3_, 2 mM CaCl_2_, 1 mM MgCl_2_, 25 mM glucose) and then placed with the dorsal side facing up in a bath of aCSF bubbled with oxygen. Crystals of rhodamine dextran (Molecular probes, Eugene, OR) were applied to a unilateral cut at L4. The preparations were then incubated in oxygenated aCSF solution for 12–15 hr at room temperature. Spinal cords were then fixed in 4% PFA, infiltrated with 20% sucrose and cryosectioned into 50 µm thick sections. Sections were mounted in Fluoromount-G (Southern Biotech, Birmingham, AL) and imaged at L2 to detect axonal crossing using an epifluorescence microscope (Zeiss Axio). The experiment was repeated with four animals per genotype. The mean labeling intensity was quantified using NIH ImageJ software.

### Immunohistochemistry

50 µm thick, cryostat-cut spinal cord sections at L2 level were subjected to immunohistochemistry for ephrinB3 staining. Briefly, the sections were blocked in 3% BSA with 0.1% Triton X-100 for 1 hr. The sections were then incubated overnight with anti-ephrinB3 antibody at 4°C. Alexa 488 anti-rabbit secondary antibody was added for 2 hr. After 3 washes with 1× PBS, the sections were mounted in Vectashield mounting medium with DAPI (Vector labs, Burlingame, CA).

### Primary Cell Culture and Growth Cone Collapse Assay

Cortical neuron cultures were prepared from the developing motor corticies of P0 mouse pups or embryonic day 18 (E18) rat embryos and plated on coverslips coated with PDL/Laminin (BD Biosciences) in Neurobasal A supplemented with B27 supplement, 200 mM L-glutamine and penicillin/streptomycin. After 24 hrs, the medium was changed to Neurobasal A (Gibco, Grand island, NY) supplemented with astrocyte-conditioned medium (Ratio 3∶1). To inhibit RhoA/ROCK signaling, rat cortical neurons were treated with 20 µM of the ROCK inhibitor Y-27632 (Tocris, Ellisville, MO) or vehicle for 1 hr. At 2 days in vitro (DIV2), the cortical neuron cultures were stimulated with 2 µg/ml of preclusted Fc (control) or ephrinB3-Fc (R&D systems, Inc, Minneapolis, MN) for 30 minutes. Fc and ephrinB3-Fc were preclusterd with an anti-Fc antibody (Jackson ImmunoResearch) in a 5∶1 molar ratio at room temperature for 1 hr as previously described [5], [27]. The cells were fixed with 4% PFA for 15 min and processed for immunostaining to visualize F-actin and microtubules. The neurons were imaged and analyzed for growth cone collapse in a blinded manner. Growth cones with no filopodia or lamellipodia were scored as collapsed. Approximately 200 neurons were analyzed per condition for each experiment and the experiment was repeated 3 independent times.

### Immunofluorescence Staining

Primary cortical neuronal cultures were fixed with 4% PFA for 15 min after the treatment at specified time points. The coverslips were washed five times with 1× PBS and blocked with the blocking solution (15% goat serum, 0.1% Triton ×100, 5% BSA in 1× PBS). Neurons were then incubated overnight with anti-acetylated tubulin antibody. After 3 washes the coverslips were incubated with Alexa 488-conjugated secondary antibody and Alexa 594-phalloidin (Molecular Probes, Eugene, OR). The cells were imaged using a Zeiss epifluorescence microscope.

### Statistical Analysis

All statistical parameters were calculated using KaleidaGraph software (Synergy Software, Reading, PA). Student’s t-test or analysis of variance (ANOVA) followed by Tukey's or Student-Newman-Keuls post-test were used to determine statistical significance. P values less than 0.001 were considered significantly different.

## Results

### Conditional Ablation of RhoA in Neuroprogenitor Cells

RhoA is widely expressed in the developing brain and spinal cord (Fig. S1). To determine the role of RhoA in the developing CNS, we generated a conditional knock-out allele of the *RhoA* gene (*RhoA^fl/fl^*) that has two loxP sites flanking exon 3 and part of intron 3 (Fig. 1A). By crossing the *RhoA^fl/fl^* mice with Nestin-Cre mice, which express Cre recombinase in neuroprogenitor cells, we ablated RhoA from the developing CNS (Fig. 1B). Western blot analysis of adult mouse brain lysates confirmed that RhoA protein levels were significantly reduced in *RhoA^+/fl^; Nestin-Cre* (hereafter referred to as RhoA het) mice, and were undetectable in *RhoA^fl/fl^; Nestin-Cre* (RhoA cKO) mice compared to *RhoA^fl/fl^* (control) mice (Fig. 1C). Loss of RhoA expression was evident in the brains of RhoA cKO mice at postnatal day 0 (P0) (Fig. 2A, B) and in the spinal cord at P5 (Fig. 2C, D). Deletion of RhoA did not affect the expression or activity of the Rho GTPase Rac1 (data not shown), but did cause a compensatory increase in the expression of the other Rho isoforms, RhoB and RhoC (Figs. 2 A–D), as has previously been reported in other systems [28]–[30].

### RhoA cKO Mice Exhibit Locomotor Defects

The RhoA cKO mice were born at the expected Mendelian ratios, survived into adulthood, and showed no obvious signs of morbidity. However, these mice exhibited a rabbit-like hopping gait that differed markedly from the normal gait displayed by control littermates (Fig. 3A–C) or RhoA het mice (data not shown). While control mice moved their right and left limbs in alternation, RhoA cKO mice synchronously moved their forelimbs and then their hindlimbs in parallel (Movie S1, S2). This locomotor defect could be detected as early as P0 (data not shown) and was maintained throughout adulthood. Notably, the RhoA cKO mice phenocopy locomotor defects observed in mice lacking ephrinB3, EphA4 or EphA4 forward signaling [3]–[5], [27].

### Aberrant Midline Crossing of Corticospinal Tract Axons in RhoA cKO Mice

The hopping gait displayed by *ephrinB3^−/−^* and *EphA4^−/−^* mice is accompanied by an inappropriate midline crossing of corticospinal tract fibers [4], [6]. The CST is a bundle of descending axons that control fine motor function by connecting corticospinal neurons in the motor cortex with primary motor neurons and interneurons in the spinal cord. CST fibers normally decussate at the caudal end of the medulla and, in rodents, project down the spinal cord at the base of the dorsal funiculi, and then branch off collateral fibers to innervate neurons ipsilateral to the tracts [31]. Most CST axons are prevented from crossing the spinal cord midline to the contralateral side by ephrinB3, which forms a repulsive midline barrier [4]. To determine the role of RhoA in CST pathfinding, we examined CST projections in control, RhoA het, and RhoA cKO mice. The anterograde tracer biotinylated dextran amine (BDA) was unilaterally injected into the right sensorimotor cortex to selectively label the left CST axons (Fig. 4A). In control and RhoA het mice, these CST fibers predominantly projected into the gray matter ipsilateral to the labeled CST (Fig. 4C). In contrast, many CST axons in the RhoA cKO mice appeared to have aberrantly crossed the midline and projected into the gray matter on the side contralateral to the labeled CST (Fig. 4C). Quantification of this data showed that the number of CST collaterals inappropriately projecting into the contralateral side of the spinal cord gray matter was significantly greater in the RhoA cKO than in control and RhoA het mice (Fig. 4D). These results demonstrate that RhoA is necessary for the proper pathfinding of CST projections.

### Abnormal Dorsal Funiculus Morphology in RhoA cKO Mice

CST fibers normally descend down the spinal cord through the ventromedial part of the dorsal funiculus. Histological analysis of lumbar spinal cord cross sections revealed that the morphology of the dorsal funiculus in RhoA cKO mice was also abnormal. Compared to control and RhoA het mice, the dorsal funiculus in RhoA cKO mice was wider and shallower and was accompanied by an extended gray matter at the spinal cord midline (Fig. 5 A,B,C). This result suggests that RhoA plays a role in regulating dorsal funiculus formation.

### Aberrant Axonal Projections of Spinal Interneurons in RhoA cKO Mice

The synchronous limb locomotion defect observed in the *RhoA* cKO mice points to dysfunction in the central pattern generator (CPG). The CPG is a neuronal circuit in the spinal cord that provides bilateral control over normal limb alternation that underlies walking [32]. EphrinB3-induced EphA4 signaling has been shown to regulate CPG development by detering the axons of ipsilaterally projecting excitatory spinal interneurons from inappropriately crossing the midline, preventing aberrant innervation of the contralateral CPG [6]. To determine whether RhoA plays a role in this process, we performed unilateral injections of rhodamine dextran into the lumbar spinal cord at level L4 and examined retrogradely labeled axonal projections in transverse sections at L2 (Fig. 5D). As expected, the control mice showed labeled fibers projecting primarily to the ipsilateral side of the spinal cord, whereas in the RhoA cKO mice, many fibers aberrantly crossed over the midline to the contralateral side at L2 (Fig. 5E). Quantiatitive analysis of the mean labeling intensity near the midline on the contralateral side further confirmed that RhoA-deficient mice exhibit a significant increase in midline crossing: 0.31±0.12 in control mice compared to 29.40±2.35 in RhoA cKO mice (P<0.001; N = 4). This result indicates that similar to the CST, RhoA is required for restricting midline crossing of interneurons in the spinal cord.

### RhoA is Required for EphrinB3-induced Growth Cone Collapse

**Figure 6:**
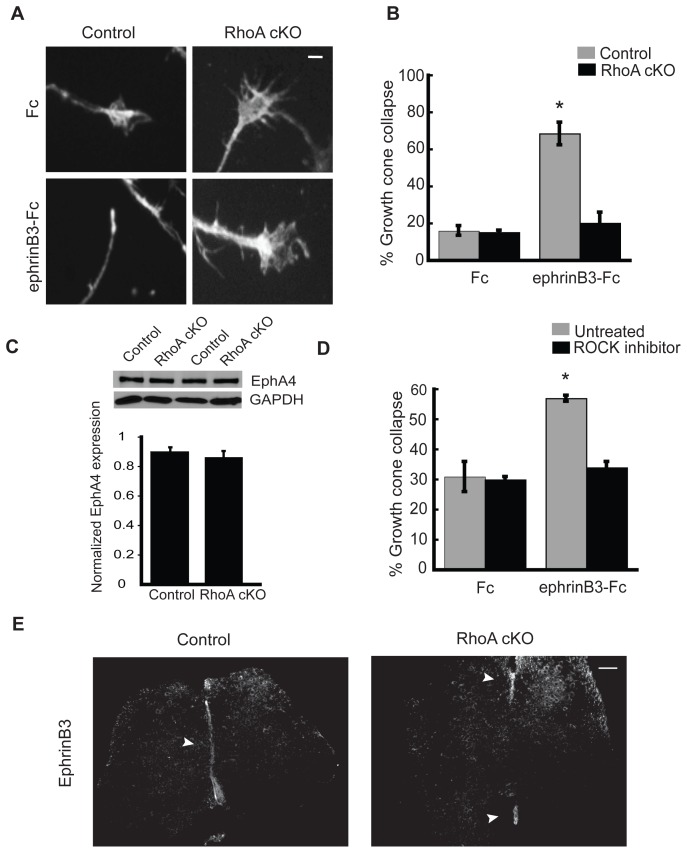
Loss of RhoA inhibits ephrinB3-induced growth cone collapse and disrupts ephrinB3 midline expression. (A) Representative images of growth cones from control and RhoA cKO cortical neurons after a 30 min incubation with preclustered ephrinB3-Fc or Fc alone. Scale bar:10 µm. (B) Quantification of the growth cone collapse response of control and RhoA cKO neurons treated with ephrinB3-Fc or Fc (*p<0.001, ANOVA followed by the Tukey test). (C) Cortical neurons from RhoA cKO mice express EphA4 at levels similar to control neurons. (D) Percentage of ephrinB3-induced growth cone collapse in rat cortical neurons pretreated or not with the ROCK inhibitor Y-27632 (*p<0.001, ANOVA followed by the Tukey test). (E) Expression of ephrinB3 is lost from the midline at L2 in RhoA cKO mice. Arrowheads show ephrinB3 positive staining in the spinal cord of P5 mice. Scale bar: 10 µm. Three sections per animal and N = 3 animals were analyzed per genotype.

A repulsive ephrinB3 midline barrier normally prevents EphA4-expressing CST and spinal interneuron axons from inappropriately crossing the spinal cord midline [4]. To determine whether RhoA-deficient neurons have lost their ability to respond to ephrinB3, we utilized a growth cone collapse assay to image repulsive EphA4 receptor signaling responses [20], [22], [33]. P0 mouse cortical neurons from the presumptive motor cortex of control and RhoA cKO mice were cultured for 2 days in vitro (DIV) and then subjected to preclustered ephrinB3-Fc or Fc (control) for 30 minutes. The neurons were then fixed and stained with phalloidin to visualize filamentous actin (F-actin) in the growth cones. In response to ephrinB3 stimulation, control neurons exhibited growth cone collapse, whereas axons from RhoA cKO neurons remained uncollapsed after 30 minutes of treatment (Fig. 6A, B). This difference was not due to loss of EphA4 expression, since EphA4 protein levels were unchanged in cortical neurons from control and RhoA cKO mice (Fig. 6C,). EphrinB3-induced growth cone collapse was similarly blocked in embryonic day 18 (E18) rat cortical neurons when they were preincubated with Y-27632, an inhibitor for ROCK, the major downstream effector of RhoA (Fig. 6D). Together, these findings indicate that RhoA signaling is necessary for neurons to properly respond to the repulsive effects of ephrinB3.

### Absence of EphrinB3 in the Spinal Cord Midline in RhoA cKO Mice

Since RhoA has been reported to regulate spinal cord neuroepithelium organization [34], we also investigated the possibility that the ephrinB3 repulsive cue itself could be lost from the midline. We stained L2 level spinal cord sections from P5 control and RhoA cKO mice with anti-ephrinB3 antibody and observed that indeed ephrinB3 expression was disrupted in RhoA cKO mice (Fig. 6E). EphrinB3 staining in RhoA cKO mice was restricted to the dorsal and ventral midline areas and was absent from the center of the spinal cord, in contrast to control animals (Fig. 6E). This absence of a respulsive ephrinB3 midline barrier likely allows EphA4-positive CST and spinal interneuron axons to inappropriately cross over to the contralateral side of the spinal cord, resulting in abnormal formation of locomotor circuits.

## Discussion

**Figure 7:**
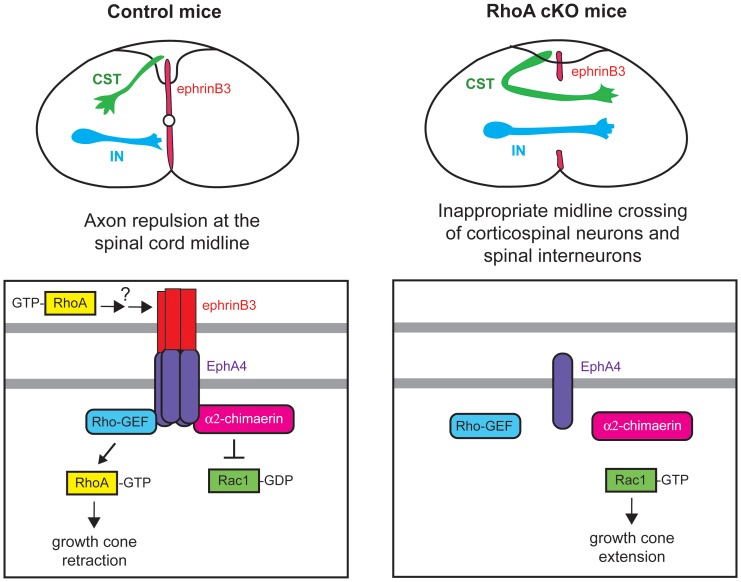
Proposed model to illustrate the role of RhoA in ephrinB3/EphA4-dependent locomotor circuit assembly. In control mice, when EphA4-expressing CST and spinal interneuron (IN) axons encounter ephrinB3 at the spinal cord midline, they are repulsed due to the initiation of signaling pathways that involve RhoA activation and Rac1 inhibition. Loss of RhoA causes aberrant midline crossing of CST and spinal IN axons due to a failure of neurons to retract their axons and/or the absence of ephrinB3 expression at the midline.

In the developing spinal cord, ephrinB3 functions as a repulsive midline barrier that prevents EphA4-expressing CST and spinal interneuron axons from crossing the midline, ensuring proper formation of locomotor circuits (Fig. 7). In this study, we show that the small GTPase RhoA is required for ephrinB3/EphA4-dependent locomotor circuit formation. Deletion of RhoA from neural progenitor cells results in mice that exhibit a rabbit-like hopping gait with synchronous limb movements that resemble mice lacking ephrinB3 or EphA4. Consistent with this locomotor defect, we found that CST and spinal interneuron projections from RhoA-deficient mice aberrantly cross the spinal cord midline, resulting in inappropriate bilateral innervation. Futhermore, we determined that loss of RhoA blocks both ephrinB3-induced growth cone collapse of axons from cortical neurons and disrupts ephrinB3 expression at the spinal cord midline. These results demonstrate that RhoA is necessary for the ephrinB3/EphA4-dependent development of cortical and spinal motor circuits that control locomotor behavior.

How does RhoA regulate the formation of cortical and spinal locomotor circuits? When EphA4-positive CST and spinal interneuron axons encounter ephrinB3 at the spinal cord midline, EphA4 is activated, triggering actin cytoskeletal remodeling that drives growth cone collapse and axon repulsion. The mechanism by which activated EphA4 transduces signals to the actin cytoskeleton to control repulsive axon guidance during locomotor circuit formation is not fully understood. Our results suggest that RhoA and its downstream target kinase ROCK are required for neurons to properly respond to the repulsive effects of ephrinB3, since ephrinB3 fails to induce growth cone collapse when RhoA is deleted or ROCK is inhibited. The increased expression of the highly related proteins RhoB and RhoC is not able to fully compensate for this defect in RhoA-deficient neurons. RhoA signaling is thought to promote growth cone collapse and axon retraction by modulating F-actin orgnaization and myosin II activity [35]. EphA4 may induce RhoA activation in response to ephrinB3 via the guanine nucleotide exchange factor (GEF) Ephexin1, which has been demonstrated to function downstream of EphA4 in repulsive axon guidance [16], [17]. However, Ephexin1 KO mice do not exhibit a rabbit-like hopping gait [17], suggesting that other RhoA-GEFs may act downstream of EphA4 in regulating cortical and spinal motor circuit assembly.

Recently, mice lacking α2-Chimaerin were also reported to display a rabbit-like hopping gait [20]–[22]. α2-Chimaerin is a Rac-specific GTPase-activating protein (GAP) that binds to activated EphA4 receptors and mediates ephrinB3-induced growth cone collapse by inhibiting Rac1 [33]. Loss of α2-Chimaerin leads to the hyperactivation of Rac1, which causes aberrant midline crossing of CST and spinal interneuron axons that fail to respect the repulsive ephrinB3 barrier [22]. Since Rac1 and RhoA are known to have opposing effects on cytoskeletal remodeling and axon extension, it is not entirely surprising that deletion of RhoA phenocopies loss of a RacGAP. EphrinB3/EphA4-dependent axon guidance decisions and subsequent locomotor circuit formation likely require a dynamic balance between RhoA and Rac1 activities.

Our results also indicate that RhoA regulates ephrinB3 expression at the spinal cord midline, which is consistent with a recent report from the Yoshida lab [36]. The authors showed that deletion of RhoA from neural progenitors in the spinal cord causes disorganization of the spinal cord neuroepithelium, resulting in loss of ephrinB3 expression from the midline and defects in neuronal projections and left-right motor behavior. The authors did not, however, explore the cell autonomous role of RhoA in EphA4-dependent axon guidance. RhoA is thought to control neuroepithelial organization in the developing spinal cord by regulating adherens junctions [34], [36]. Deletion of RhoA results in loss of ephrinB3 expression from the center but not the dorsal and ventral areas of the spinal cord midline (Fig. 6E) [36], suggesting that RhoA helps establish ephrinB3 midline expression in the central spinal cord. However, the precise mechanism by which RhoA regulates ephrinB3 expression at the midline is not known and requires further investigation. In summary, it is clear that RhoA prevents CST and spinal interneuron projections from inappropriately crossing the midline by both regulating ephrinB3 midline expression and by promoting growth cone collapse and axon retraction by modulating F-actin organization and myosin II activity. These functions of RhoA are essential for the proper assembly of cortical and spinal cord neural circuits and for appropriate locomotor behavior.

## Supporting Information

Figure S1 **RhoA expression in P4 mouse brain and spinal cord.** *In situ* hybridization was used to detect RhoA message in (A) sagittal sections of P4 mouse brain and (B) transverse sections of P4 mouse spinal cord. These images, couresy of the Allen Developing Mouse Brain Atlas (http://developingmouse.brain-map.org) and the Allen Spinal Cord Atlas (http://mousespinal.brain-map.org/), indicate that RhoA is widely expressed throughout the developing brain and spinal cord.
(TIF)

Movie S1 **Normal gait in control mouse.** Control mice use alternate limb movements during walking.
(WMV)

Movie S2 **Hopping gait in RhoA cKO mouse.** RhoA cKO mice use their forelimbs and hindlimbs sychronously during walking.
(WMV)
