# Supplementary figures and images for: The Small GTPase RhoA Is Required for Proper Locomotor Circuit Assembly
Source: PLoS One. 2013 Jun 25;8(6):e67015. doi: 10.1371/journal.pone.0067015 (PMC3692541; doi:10.1371/journal.pone.0067015)

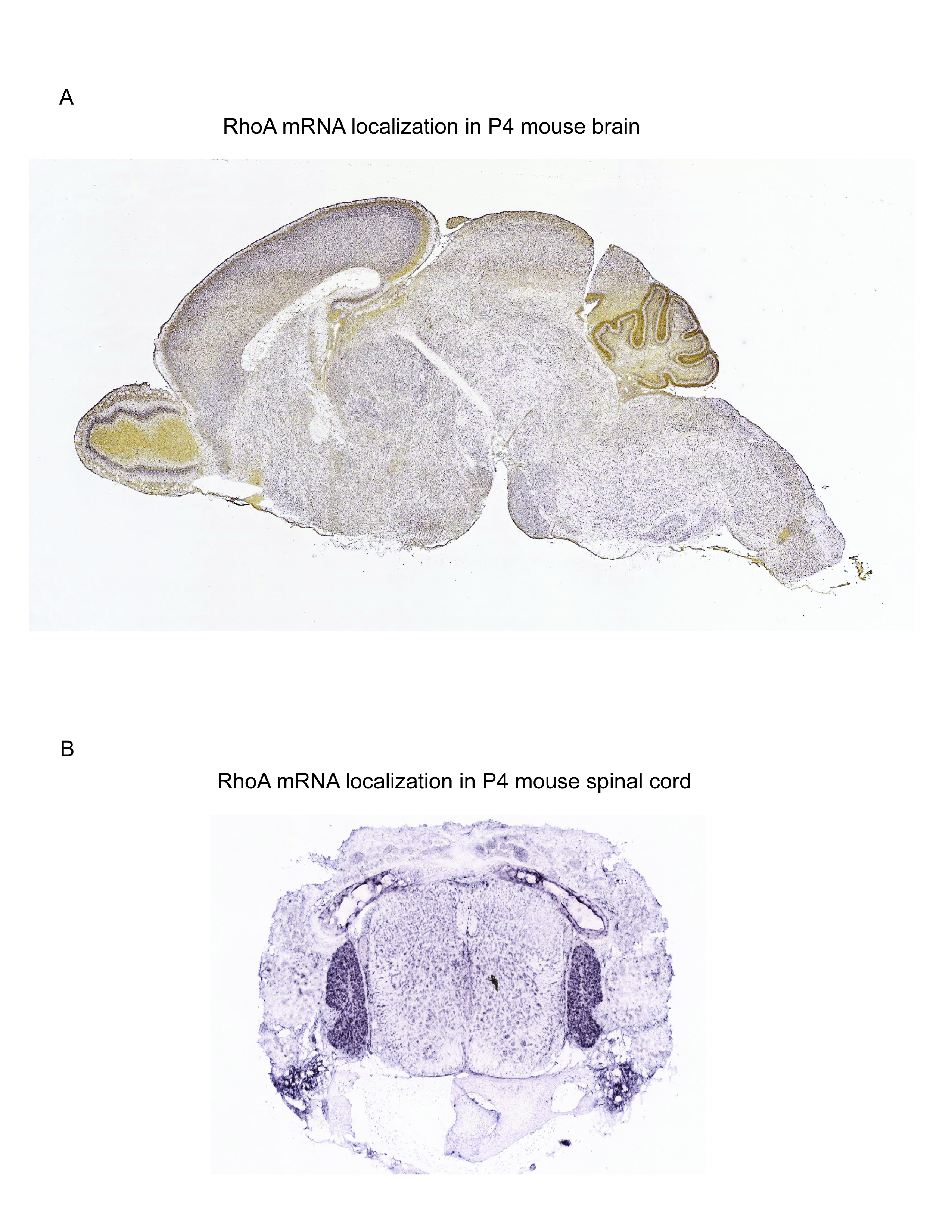

Supplement: Figure S1 — RhoA expression in P4 mouse brain and spinal cord. In situ hybridization was used to detect RhoA message in (A) sagittal sections of P4 mouse brain and (B) transverse sections of P4 mouse spinal cord. These images, couresy of the Allen Developing Mouse Brain Atlas (http://developingmouse.brain-map.org) and the Allen Spinal Cord Atlas (http://mousespinal.brain-map.org/), indicate that RhoA is widely expressed throughout the developing brain and spinal cord. (TIF) [file pone.0067015.s001.tif]
